# Evaluating the Effectiveness of the Ponseti Technique in Treating Idiopathic Clubfoot: Long-Term Outcomes From an Indian Tertiary Care Centre

**DOI:** 10.7759/cureus.100658

**Published:** 2026-01-03

**Authors:** Amrit Goyal, Sudhanshu Agarwal, Mayur Gupta, Rajat Kapoor, Mohit Mittal, Avnish K Mishra

**Affiliations:** 1 Orthopaedics, Sarojini Naidu Medical College, Agra, Agra, IND; 2 Orthopaedics, Maharshi Vishwamitra Autonomous State Medical College, Ghazipur, IND

**Keywords:** bracing, clubfoot, ctev, idiopathic, ponseti method

## Abstract

Introduction: Congenital talipes equinovarus (CTEV) is a common pediatric musculoskeletal deformity, with a male predominance. The Ponseti method, which involves serial casting and bracing, has become the global standard because of its high success rate and reduced need for surgery. This study aimed to evaluate the long-term effectiveness of the Ponseti method in managing idiopathic clubfoot at a tertiary care centre in India.

Materials and methods: This retrospective study analyzed patients with idiopathic clubfoot treated with the Ponseti method at our institute. A total of 331 patients were included, of whom 219 (66%) had bilateral involvement and 112 (34%) had unilateral clubfoot, accounting for 550 affected feet. The inclusion criteria were the absence of prior surgery and the availability of complete records. Treatment included weekly casting, Achilles tenotomy, and bracing with Denis-Browne splints. Outcomes were assessed using Pirani scores.

Results: In our study, 71.29% (n = 236) were males and 28.7% (n = 95) were females. The mean age at presentation was 1.20 months. A total of 94.7% (n = 521) of the feet achieved full correction, with Pirani scores decreasing from 4.36 to 0.43 (p < 0.0001). Tenotomy was required in 77.20% (n = 425) of feet for equinus correction. The average number of casts required for correction was 5.18. Residual deformities were observed in 5.3% (n = 29) of feet. Poor compliance with the bracing protocol was the main reason for these residual deformities in our study.

Conclusion: The Ponseti method remains a highly effective and reliable technique for managing idiopathic clubfoot when applied with adherence to the protocol and adequate caregiver support. Our findings not only reaffirm its global effectiveness but also highlight the importance of early treatment initiation, which reduces the number of casts required and improves outcomes compared with later interventions. The Ponseti technique markedly decreased the need for surgical correction of CTEV in our setting. Sustained bracing and follow-up are critical for preventing relapse. Future research should focus on identifying barriers to brace compliance and evaluating strategies to improve long-term outcomes.

## Introduction

Congenital talipes equinovarus (CTEV), commonly known as clubfoot, is a prevalent musculoskeletal deformity of the lower limbs. It is characterized by a complex combination of foot abnormalities, including cavus, varus, adductus, and equinus deformities [1]. The primary objective of treatment is to achieve a functional, pain-free, plantigrade foot with adequate mobility and to prevent recurrence.

The global incidence of CTEVs is estimated to be 1-2 cases per 1,000 live births. This condition is more prevalent in males, occurring approximately three times more frequently than in females. In half of the cases, the deformity affected both feet. Although CTEV can be associated with neuromuscular or syndromic conditions, such as myelodysplasia and arthrogryposis, the majority of cases are idiopathic and present without any underlying pathology [1].

Numerous etiological hypotheses for congenital clubfoot have been proposed, subsequently abandoned, and later revisited by successive generations of researchers. Among the earliest theories was the mechanical theory, originally described by Hippocrates, which posited that clubfoot results from elevated intrauterine pressure during pregnancy. This hypothesis was later refuted due to the lack of an increased incidence under conditions of presumed uterine crowding, such as multiple gestations, macrosomia, or polyhydramnios.

Several authors have proposed histological mechanisms to explain the development of clubfoot. Loren et al. [2] demonstrated that abnormal histology of the peroneus brevis correlates with an increased risk of relapse. Irani and Sherman [3] suggested that a primary germ-plasm defect is the underlying cause. Anatomical abnormalities have also been implicated; Ippolito [4] reported medial angulation of the talar neck along with medial tilting and rotation of the talar body.

CTEV is primarily a clinical diagnosis, usually apparent at birth or detected antenatally using ultrasonography. On physical examination, the affected foot is typically smaller, with a deep medial crease and an accentuated longitudinal arch. Gentle manipulation reveals the degree of rigidity, which distinguishes true CTEV from positional or postural deformities. Although imaging is not routinely required in neonates, radiography or ultrasonography may be employed in atypical cases or to assist with the surgical planning.

Over the years, various nonsurgical approaches have been explored for treating CTEV. One of the earliest known efforts dates back to 1836, when Guerin used plaster casts to correct deformities. Kite later introduced a method that focused on gradual correction through gentle manipulation followed by casting [5]. A major turning point occurred in the mid-1900s when Dr. Ignacio Ponseti developed a structured approach using a series of casts and braces. This method, which is now widely adopted worldwide, has shown excellent results and has significantly reduced the need for surgery in most cases [6].

We have been practicing the Ponseti method to correct CTEV at our institute for the past 15 years and have witnessed a decline in the number of surgical interventions. This study aimed to assess the effectiveness of the Ponseti method in the management of idiopathic clubfoot in our local population, focusing on clinical outcomes and treatment success rates.

## Materials and methods

This retrospective study was conducted at Sarojini Naidu Medical College, Agra, India, after approval from the Institutional Ethics Committee of our institute (letter number: SNMC/IEC/DHR/2025/134). The study included patients enrolled in the clubfoot clinic of the Outpatient Department of Orthopaedics at our institute, who had been treated using the Ponseti method since 2013 and had voluntarily provided written informed consent to participate in the study. Medical records were examined to evaluate the treatment outcomes. Each patient was followed up for at least four years to assess residual deformities, pain, and range of motion of the affected feet. During follow-up visits, clinical evaluations were performed to determine the long-term success of the Ponseti technique. The Pirani scores recorded before and after treatment were compared with the patient’s current clinical status.

All patient evaluations and data analyses were conducted by certified orthopedic surgeons, each with over five years of clinical experience in managing congenital foot pathologies such as CTEV. Their training in the Ponseti method and familiarity with standardised clinical and baropodometric protocols ensured consistency and reliability of the assessments.

The inclusion criteria were a diagnosed case of idiopathic clubfoot, no prior surgical intervention, and available follow-up data of at least four years with Pirani scores. Patients with syndromic or neuromuscular clubfoot and those with incomplete records were excluded.

All patients were treated using the Ponseti method, which is widely regarded as the gold standard for conservative management of idiopathic clubfoot. This technique involves a sequence of serial manipulations and plaster cast applications aimed at gradually correcting the deformities in a specific order: cavus, adductus, varus, and finally equinus. The severity of each clubfoot was evaluated using the Pirani scoring system [7] at the time of initial presentation and recorded at each follow-up visit to track the progress and effectiveness of the interventions. The score consists of six clinical parameters: three for the hindfoot (posterior crease, emptiness of the heel, and rigid equinus), and three for the midfoot (medial crease, curvature of the lateral border, and palpability of the lateral head of talus). Each parameter is scored as 0 (normal), 0.5 (moderately abnormal), or 1 (severely abnormal). The total score ranges from 0 to 6, with higher scores indicating more severe deformity.

After correction was achieved with weekly plaster casts, Achilles tenotomy was performed when passive dorsiflexion of the ankle remained <15° despite adequate abduction of the foot, as stated in the original article by Ponseti [8]. This minimally invasive procedure was performed on an outpatient basis at our institute under local anesthesia in the operating theatre by a dedicated team trained to ensure both safety and efficiency. This step enabled the heel to dorsiflex to the corrected position. After tenotomy, the foot was placed in a final cast for approximately three weeks to support healing and allow for tendon regeneration.

Following cast removal, a foot abduction brace, commonly referred to as the Denis Browne abduction brace, was introduced to maintain the corrected foot alignment and prevent recurrence. The Denis Browne abduction brace was applied with the feet externally rotated to 60-70° on the affected side and 30-40° on the unaffected side, with the bar length equal to the child’s shoulder width. The brace maintained 10-15° ankle dorsiflexion. Initially, the brace was worn full-time (23 hours/day) for two to three months, followed by the use of CTEV corrective shoes during the day and continued nighttime use of the Denis Browne brace for four to five years.

Brace compliance was assessed during each follow-up visit based on caregiver interviews and documentation in hospital records. Children wearing the brace according to the prescribed schedule were considered compliant, whereas those with irregular or discontinued brace use were categorized as non-compliant.

Demographic variables such as age, sex, and laterality were recorded on a per-patient basis. Clinical severity was assessed using the Pirani scoring system, and treatment-related outcomes, including the number of casts, need for tenotomy, and final correction, were analyzed on a per-foot basis.

Statistical analysis was performed using IBM SPSS Statistics for Windows, Version 22 (Released 2013; IBM Corp., Armonk, New York, United States). Descriptive statistics were used to summarise demographic and clinical characteristics, and values were presented as mean ± standard deviation (SD) or percentages where applicable. Pre- and post-treatment Pirani scores were compared using a paired t-test. The association between age at initiation of casting and the number of casts required for correction was evaluated using one-way analysis of variance (ANOVA). A p-value of <0.05 was considered statistically significant for all comparisons.

## Results

**Figure 1:**
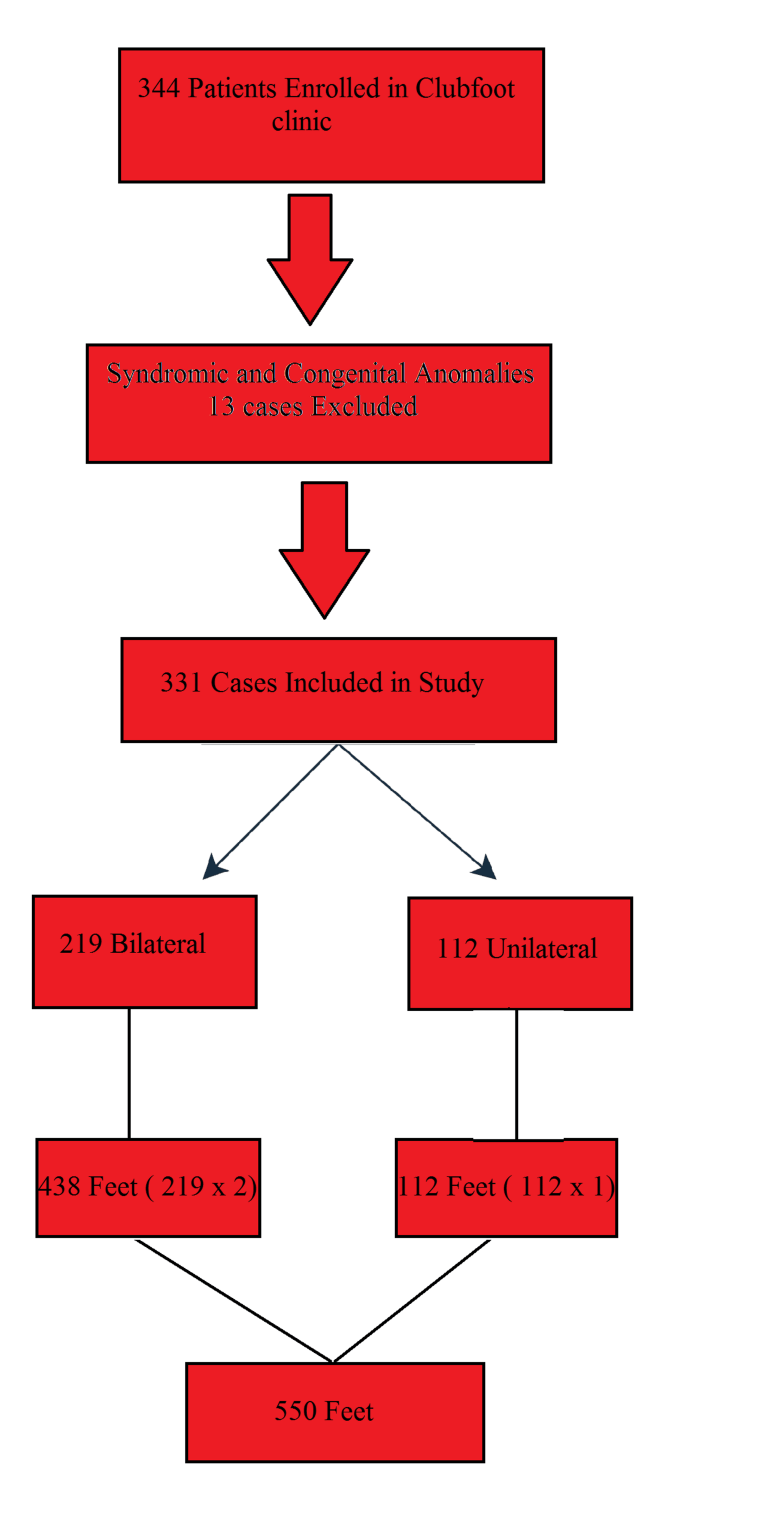
Patient Enrollment Flowchart

The patient enrollment flowchart is shown in Figure 1. A total of 331 patients enrolled in the Clubfoot Clinic were included in the study, accounting for 550 affected feet. Bilateral clubfoot was present in 219 patients (66%), contributing 438 feet, while 112 patients (34%) had unilateral involvement, contributing 112 feet. Our Clubfoot Clinic records initially included 344 children. Among them, 13 were identified as having congenital anomalies or were classified as syndromic cases. Although these children were initially managed using the Ponseti method, similar to those with idiopathic CTEV, their outcomes were poor, with a high relapse rate. Consequently, these patients were excluded from the main study population. Syndromic cases were treated surgically. However, even with surgical intervention, the results were unsatisfactory.

All patients were systematically evaluated and treated according to standardised protocols. Among the total cohort, 71.29% (n = 236) were male, and 28.7% (n = 95) were female, reflecting a male-to-female ratio of approximately 2.48:1, which is consistent with the established male predominance of idiopathic clubfoot.

The mean age at the time of first presentation was 1.20 months, with ages ranging from seven days to four months. This age distribution underscores the variability in presentation and diagnosis, particularly in regions with limited access to early orthopaedic care. Notably, a positive family history of CTEV was observed in only 3.02% (N = 10) of the cases.

On the basis of clinical history and previous treatment status, the study population was categorised into three distinct groups. The majority of patients, comprising 75% (n = 413), presented with primary idiopathic clubfoot and had received no prior intervention before enrolment. Relapse clubfoot accounted for 17% (n = 93) of cases and included children who had achieved initial correction following treatment but subsequently developed a recurrence of the deformity. The remaining 8% (n = 44) were classified as recurrent clubfoot, referring to patients who had undergone earlier corrective treatment but experienced repeated failures in maintaining alignment over time.

Relapse was defined as the reappearance of deformity after successful initial correction, whereas recurrent cases were characterised by persistent inability to sustain correction despite multiple treatment attempts. Both relapse and recurrent groups were treated using serial manipulation and casting according to the Ponseti technique.

At the final follow-up assessment, no statistically significant difference was observed in the mean Pirani scores among the primary, relapse, and recurrent groups (Table 1), suggesting that comparable clinical outcomes were achieved irrespective of the initial disease presentation.

**Table 1:** Comparison of Final Pirani Scores Between Primary, Relapse, and Recurrent Cases One-way ANOVA showed no statistically significant difference in final Pirani scores between the three groups (F = 1.77, p = 0.18). N represents the number of feet. ANOVA: analysis of variance; SD: standard deviation

Group	N (%)	Mean Final Pirani Score	SD
Primary	413 (75)	0.42	0.14
Relapse	93 (17)	0.45	0.16
Recurrent	44 (8)	0.44	0.15

All cases underwent correction using a standardised Ponseti regimen, and follow-up assessments were performed in accordance with established clinical guidelines. The mean follow-up duration was 4.81 years.

Currently, 94.7% of feet (n = 521) achieved complete correction, indicating high success rates with the standardised protocol. The average Pirani score at the start of treatment was 4.36 (range: 3-5), which decreased to 0.43 (range: 0-0.5) after treatment. A paired t-test was applied to compare Pirani scores before and after treatment, and a p-value of < 0.0001 was obtained, as shown in Table 2.

**Table 2:** Pirani Scores Before and After Treatment A paired t-test was used to compare pre-treatment and post-treatment Pirani scores. N represents the number of feet. SD: standard deviation

Pirani Score	N (%)	Mean	SD	t-value	p-value
Before treatment	550 (100)	4.36	0.90	-110.78	<0.0001
After treatment	550 (100)	0.43	0.15		

The difference between the Pirani scores before and after treatment was highly statistically significant, indicating that the Ponseti method led to a significant reduction in deformity scores.

**Figure 2:**
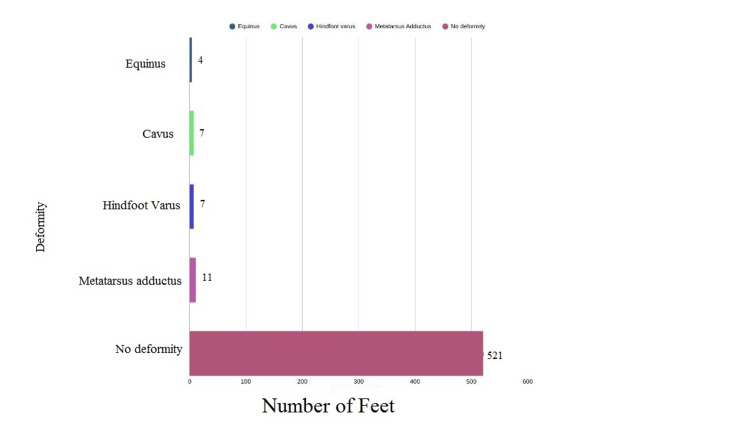
Clinical Outcomes at the Time of Last Follow-Up

The clinical outcomes at the time of the study are summarised in Figure 2. Minor residual deformities were observed in 29 feet (5.3%). Poor brace compliance was documented in the majority of these cases and was identified as the most frequent factor associated with residual or recurrent deformity. The deformities were recorded as follows: 11 feet of metatarsus adductus, seven of hindfoot varus, seven of cavus, and four of equinus. These findings underscore the importance of adherence to post-correction protocols and highlight areas for further education and support to improve long-term outcomes of CTEV management.

The number of casts required to achieve full correction ranged from four to seven, with a mean of approximately 5.18. A strong positive correlation between age at first casting and number of casts was observed after applying a single-factor ANOVA test (Table 3).

**Table 3:** Number of Casts in Relation to Age of First Visit (Months) One-way ANOVA showed a statistically significant difference among the three age groups (F = 1268.05, p < 0.0001). N represents the number of feet. ANOVA: analysis of variance; SD: standard deviation

Age at First Visit (Months)	N	Mean Number of Casts	SD
≤1.2	463	4.98	0.14
1.3-2.4	23	5.35	0.49
>2.4	64	6.55	0.50

The development of cast-related pressure sores was observed in a few cases (N = 23). These sores resolved after a treatment gap of seven days and a course of oral antibiotics. During this phase, the mother was trained to execute regular phased manipulation of the baby’s feet.

The initial deformity components (cavus, adduction, and varus) generally responded well to gentle manipulation, followed by weekly serial casting. However, the correction of equinus deformity often requires additional interventions. Percutaneous Achilles tenotomy was performed in 77% of feet (n = 425) to achieve complete correction of the equinus component.

## Discussion

The primary goal of treating congenital clubfoot is to achieve a pain-free, plantigrade foot with a good range of motion and no calluses. Traditionally, many children with this condition have undergone surgical procedures to correct deformities. However, studies showing limited long-term success following surgery have prompted a shift in clinical practice. Orthopaedic specialists increasingly favour non-surgical interventions, particularly serial casting techniques, over operative management [9].

Initially, Kite developed a treatment protocol, but the results were not promising. Various previous studies have compared the Kite’s and Ponseti techniques, in which Ponseti’s technique had clearly outperformed Kite’s protocol for correction of CTEV [10,11]. Long-term follow-up studies of the Ponseti method, a non-invasive approach involving gentle manipulation and casting, have demonstrated favourable outcomes [12]. There is a broad consensus that nonsurgical treatment should be the first-line management for congenital clubfoot.

One of the notable benefits of this approach is that it can be administered by trained nonsurgical healthcare providers [13,14]. This allows for greater accessibility and helps reduce the demand for operating rooms, which are often in high demand in many healthcare settings.

In our study, the majority of patients were male, accounting for 71.29% of the cohort population. This male predominance is consistent with previous studies. For instance, Haft et al. [15], Saini et al. [16], and Sakale et al. [17] reported a higher incidence in males, with proportions of 65%, 71%, and 60%, respectively. These consistent observations suggest a possible sex-related predisposition to congenital CTEV in males.

In our study, the mean number of casts required to achieve full correction was 5.18. This finding is consistent with the results of several previous studies, as shown in Table 4. For instance, Morcuende et al. [18] documented an average of five casts, while Bhatiwal et al. [19] reported 5.4 casts, whereas Sakale et al. noted an average of 5.8. Our results fall within this range, further supporting the overall consistency of the casting requirements when using the Ponseti method across different populations and clinical settings.

**Table 4:** Correction Outcomes in Clubfoot Across Published Studies

Studies	Sample Size	Average Number of Casts
Morcuende et al. [18]	157 patients/256 feet	5
Bhatiwal et al. [19]	300 patients/456 feet	5.4
Sakale et al. [17]	13 patients/20 feet	5.8
Saini et al. [16]	50 patients/76 feet	6.0
Our study	331 patients/550 feet	5.18

A strong positive correlation was observed between the number of casts applied to achieve correction and age at the start of treatment. This result is consistent with those of previous studies by Vaishy et al. [20] and Mohan et al. [21]. This is because CTEV is a musculoskeletal disease, and a baby's ligaments and tendons are more elastic in the first few months of life, making it easier to stretch and reshape the foot into a corrected position gently. While the Ponseti method can still be effective in older children, they may require more casts to achieve the same correction owing to increased tissue stiffness in older children. No statistically significant difference was observed in the outcomes between early and late presenters, except for the number of casts required to achieve the correction. In the present study, the number of casts required for correction showed a significant association with the age at which treatment was initiated. Children who presented at a later age required a greater number of casts to achieve full correction. This observation was statistically significant on one-way ANOVA (F = 1268.05, p < 0.0001), reinforcing the importance of early initiation of the Ponseti method.

In our study, tenotomy was required to correct equinus in 77% of feet (n = 425). The tenotomy rates varied across studies (Table 5). Tenotomy is a minimally invasive yet highly effective intervention for addressing the equinus component of CTEV. In a recent comparative analysis, Sapienza et al. [22] reported that children with bilateral CTEV who underwent tenotomy demonstrated static and dynamic parameters similar to those of unaffected controls. Moreover, the authors observed that for certain dynamic performance measures, bilateral cases achieved superior outcomes compared with unilateral cases.

**Table 5:** Tenotomy Rates Reported Across Published Studies

Study	Sample Size	Tenotomy
Saini et al. [16]	50 patients/76 feet	87%
Bhatiwal et al. [19]	300 patients/456 feet	78%
Sakale et al. [17]	13 patients/13 feet	50%
Laaveg et al. [12]	70 patients/104 feet	90%
Our study	331 patients/550 feet	77%

In our study, 94.7% of the feet were fully corrected. Comparable correction rates for CTEV have been reported in previous studies (Table 6).

**Table 6:** Correction Outcomes With Ponseti Regimen Reported Across Studies

Study	Sample Size	Outcome (%)
Bhatiwal et al. [19]	300 patients/456 feet	97
Morcuende et al. [18]	157 patients/256 feet	95
Saini et al. [16]	50 patients/76 feet	100
Mohan et al. [21]	50 patients/76 feet	91
Laaveg et al. [12]	70 patients/104 feet	89
Our study	331 patients/550 feet	94.7

A study conducted by Pavone et al. [23] demonstrated that children with idiopathic clubfoot managed using the Ponseti method reached independent walking somewhat later than controls, but still within the expected developmental window. Furthermore, their study confirmed that language acquisition was not delayed and that long-term functional outcomes were excellent, which is consistent with our present results.

Minor residual deformities were observed in 5.3% (N = 29) of the feet. Noncompliance with bracing could be a contributing factor. This was mostly observed in children from rural areas and those with low socioeconomic status (SES). These were uniplanar deformities that did not affect the activities of daily living of the patients.

A link has been established between brace non-compliance and lower educational levels in rural regions of the United States [24]. However, currently, no data are available to evaluate the impact of cultural factors on brace adherence in India. This may be an area for further research, exploring the correlations between parental socioeconomic and educational status and brace compliance in India. We recommend long-term counselling and training of caring parents so that the child receives guided growth aimed at restoring primary foot biomechanics through physiotherapy, activity modification, resistance training, and corrective orthosis.

This study had certain limitations that should be acknowledged. First, it was a retrospective analysis, which may introduce selection bias and relies on the accuracy of the medical records. Second, although the sample size was relatively large, the study was conducted at a single tertiary care centre, which may limit the generalizability of the findings to other populations or healthcare settings. Finally, socioeconomic and cultural factors influencing brace compliance were not systematically analysed, although poor compliance was identified as a key factor for residual deformities. Future multicentre, prospective studies that include psychosocial variables are needed to strengthen the evidence.

## Conclusions

The Ponseti method offers a highly effective approach for treating clubfoot in children when performed correctly, with clear parental guidance, regular follow-up visits, and meticulous attention to detail throughout the treatment process. Our findings reaffirm its global effectiveness and highlight the importance of early initiation of treatment, which reduces the number of casts required and improves outcomes compared with later interventions. This technique has markedly reduced the need for surgical correction of CTEV in our setting. Sustained bracing and regular follow-up remain critical for preventing relapse. In regions with limited access to trained healthcare providers, increasing awareness and educating families and local caregivers about the Ponseti method play a key role in ensuring timely and effective treatment. Adherence to orthotic bracing is essential to prevent CTEV relapse and represents the most effective strategy for minimizing the prevalence of recurrent or residual deformities.
